# Low genotypic diversity and long-term ecological decline in a spatially structured seagrass population

**DOI:** 10.1038/s41598-019-54828-1

**Published:** 2019-12-05

**Authors:** Nahaa M. Alotaibi, Emma J. Kenyon, Kevan J. Cook, Luca Börger, James C. Bull

**Affiliations:** 1 Department of Biosciences, Swansea University, Swansea, Wales United Kingdom; 2 Princess Nourah bint Abdulrahman University, Riyadh, Kingdom of Saudi Arabia; 3 School of Life Sciences, University of Sussex, Brighton, England UK; 4 Natural England, Truro, England UK

**Keywords:** Plant ecology, Plant genetics, Biodiversity, Ecological genetics

## Abstract

In isolated or declining populations, viability may be compromised further by loss of genetic diversity. Therefore, it is important to understand the relationship between long-term ecological trajectories and population genetic structure. However, opportunities to combine these types of data are rare, especially in natural systems. Using an existing panel of 15 microsatellites, we estimated allelic diversity in seagrass, *Zostera marina*, at five sites around the Isles of Scilly Special Area of Conservation, UK, in 2010 and compared this to 23 years of annual ecological monitoring (1996–2018). We found low diversity and long-term declines in abundance in this relatively pristine but isolated location. Inclusion of the snapshot of genotypic, but less-so genetic, diversity improved prediction of abundance trajectories; however, this was spatial scale-dependent. Selection of the appropriate level of genetic organization and spatial scale for monitoring is, therefore, important to identify drivers of eco-evolutionary dynamics. This has implications for the use of population genetic information in conservation, management, and spatial planning.

## Introduction

Established population genetic theory predicts that increased fragmentation, as well as reduced local population size, is associated with a range of negative genetic consequences. Smaller and more isolated local populations are expected to lead to lower effective population size, increased inbreeding, as well as less allelic diversity^1,2^. However, empirical evidence across a range of major marine and terrestrial vegetation systems has resulted in equivocal support for these predictions, limiting the contribution of genetic information to conservation policy and management. In particular, the relationship between population distribution and genetic diversity has been investigated in temperate^3,4^ and tropical forests^5,6^, as well as seagrass ecosystems^7,8^ and coral reefs^9^, with mixed results.

It has become clear that it is necessary to identify the appropriate temporal and spatial scales to measure both genetic diversity and ecological indicators^10^. This should be based on knowledge of the processes and mechanisms driving eco-evolutionary dynamics governing abundance and distribution. We set out to investigate this issue using the case study of a fragmented population of seagrass, subject to long-term ecological monitoring that allows us to quantify population dynamics over appropriate temporal and spatial scales^11^.

Seagrasses are globally distributed along coastlines^12,13^, covering approximately 0.3 to 0.6 million km^2^ and are the structural basis of key coastal marine ecosystems^14^. They are terrestrial-derived, marine angiosperms that rank amongst the most productive providers of ecosystem services, encompassing supporting, provisioning, regulating, and cultural services^15,16^. Specifically, seagrass meadows support high biodiversity^17,18^ and contribute to blue carbon sequestration, nutrient cycling, erosion protection, raw materials, recreation, and nursery functions for many marine organisms^15,19–21^.

However, seagrass meadows are suffering extreme levels of damage in some parts of the world^22^ and have been undergoing large-scale declines over recent decades^23–25^. These global declines are due to a variety of factors, related either directly (as a consequence of habitat destruction, reduced water quality, physical disturbance from commercial fishing, aquaculture, and invasive species) or indirectly (e.g., through climate change) to anthropogenic activities^14,26^.

Declines in seagrass populations potentially result in increased fragmentation within meadows, as well as increased isolation between meadows^27^. These local and regional effects determine the ability of seagrass populations to survive in the face of future environmental changes^28,29^. Relatedly, isolation from surrounding populations can have profound effects on genetic variability and resulting population viability^30^. Thus understanding the genetic population structure (i.e., non-random distribution of alleles in space and time) is critical to understanding the extent to which genetic drift, dispersal, and selection contribute to long-term population decline with subsequent negative effects on ecosystem services^31–33^.

In natural monocultures, such as many seagrass ecosystems, genetic diversity may be considered similarly to species diversity, as part of the on-going diversity-stability debate^34,35^. However, average single locus genetic diversity (e.g., allele richness) may not be associated with population dynamics if neutral markers are used^36^. It is hypothesized that multi-locus genotypic diversity may provide a more reliable indicator of relevant genetic diversity as this estimates the number of genetically unique individuals, which presumably may vary in ecologically important ways^37^. As well as being associated with increased productivity^38^ and recovery^34^, higher genotypic diversity has been shown to increase diversity of associated communities^36^.

Many studies into the role of genetic diversity on population fate have focused on snapshot indicators of population state; e.g., resilience. Metrics are typically recorded within a season^34,39^ or, at best, over a couple of years^40^. While these short-term studies are valuable to test mechanistic hypotheses, natural and anthropogenic drivers may be chronic in nature, applying sustained forcing over multiple generations. It has been found that most species are not driven to extinction before genetic factors impact on them^41^. However, the relationship between genetic diversity and long-term population trajectories is less well understood.

Two sources of information are needed to tackle the challenges presented above: long-term ecological monitoring and population genetic sampling. Studies combining both these types of information are rare in any natural system. In the case of seagrasses, there are relatively few long-term studies (e.g., greater than ten years) that include appropriate temporal resolution to investigate population dynamics (but see^42–44^) and none, to our knowledge, combine ecological monitoring of seagrass distribution with data on population genetic structure. Conversely, there are numerous published studies of seagrass population genetics, but these are not formally linked to contemporary dynamics^45–48^.

The current study aims to take a first step by presenting a snapshot of population genetics alongside long-term population trajectories. Our overarching intent was to identify patterns of association between these two types of data, providing a baseline and motivation for future research into mechanisms of eco-evolutionary dynamics.

The focus of our study is the seagrass meadows around the Isles of Scilly; an archipelago situated approximately 25 miles off the southwest tip of the UK. The Isles of Scilly includes the largest continuous expanse of eelgrass, *Zostera marina*, in England and Wales and is reported to be amongst the best condition in the UK^49^. However, long-term monitoring has revealed substantial declines at this relatively isolated location^50^. In this study, we present population trajectories from 23 years of annual ecological monitoring (1996–2018) and an analysis of population genetic structure from sampling conducted in 2010. Our specific aims were to:

1. Quantify *Z. marina* population genetic variation at five long-term monitoring survey sites across the Isles of Scilly.
2. Test the hypothesis that allelic diversity is likely to be low, and inbreeding high, at this relatively isolated location.
3. Identify the spatial scale of long-term monitoring and level of genetic organization that best informs seagrass abundance trajectories.

## Results

Our goal was to assess allelic diversity in fragmented seagrass meadows subject to long-term ecological monitoring. To accomplish this, we calculated key population genetic metrics, partitioned genetic variation over spatial scales, assessed spatial independence of our five survey sites, and then present this in the context of seagrass abundance trajectories using 23 years of annual monitoring data.

### Allele scoring

A total of 96 *Z. marina* leaf samples were genotyped at 15 microsatellite loci that are highly polymorphic at other northern European sites^51^. Around the Isles of Scilly, seven loci (CL766Contig1, CL11Contig1, ZME02125, CL53Contig1, ZME06302, ZMC19062, and ZME02369) were found to be monomorphic. These seven loci were removed from all subsequent analyses, leaving a total of eight informative markers (CL559Contig1, ZMF02381, CL202Contig1, CL380Contig1, CL805Contig1, CL172Contig1, ZMC05062, ZME05315).

Amongst the remaining loci, null allele frequencies were very low. In individual samples where >10% null alleles were found; these were not consistent across loci and populations. Therefore, no further loci were discounted due to presence of null alleles. However, two loci (ZMF02381 and CL380Contig1) showed evidence of linkage to other loci (linkage disequilibrium) and were excluded from the analysis. Loci were retained in this analysis that showed no significant linkage disequilibrium (P = 0.897, Table 1). However, statistically significant positive linkage disequilibrium was observed at Old Grimsby Harbour (P = 0.008, Table 1).

**Table 1:** Estimates of genotypic diversity within five populations of *Zostera marina* around the Isles of Scilly, UK, based on six polymorphic microsatellite loci.

Site	N	MLG	eMLG	R	${\bar{{\bf{r}}}}_{{\bf{d}}}$	${\bf{P}}({\bar{{\bf{r}}}}_{{\bf{d}}})$
blt	14	10	9.6	0.69	0.055	0.078
htb	16	12	10.3	0.73	0.034	0.163
la	16	12	10.1	0.73	−0.021	0.669
ogh	13	7	7.0	0.50	0.161	0.008
wbl	14	11	10.4	0.77	−0.002	0.456
Overall	73	45	11.7	0.61	−0.017	0.897

Site: Sampling site name. N: Number of samples. MLG: Multilocus genotypes. eMLG: Estimated multilocus genotypes. R: Clonal diversity. Probability of two samples drawn at random being from the same genotype. ${\bar{{\rm{r}}}}_{{\rm{d}}}$: Linkage disequilibrium. ${\rm{P}}({\bar{{\rm{r}}}}_{{\rm{d}}})$: P-values associated with linkage disequilibrium.

### Allelic diversity

After removing 23 probable replicate clonal samples (based on P_SEX_ – see Methods), we estimated genotypic diversity from the number of multilocus genotypes (MLG) at each survey site and corresponding clonal diversity index, R. At four sites, there were 10–12 MLGs; however, at Old Grimsby Harbour there were only seven MLGs. Since we assessed a slightly reduced sample size at Old Grimsby Harbour (n = 16 rather than 20) and probable replicate samples were removed, we verified this result by comparing estimated multilocus genotypes (eMLG) at all sites based on the reduced sample size, with very similar results (Table 1). Old Grimsby Harbour was found to have substantially lower genotypic diversity (R = 0.50) than the other four survey sites (R ≥ 0.69).

An average of 1.967 alleles per locus (Allele richness, Ar) were identified across the whole of the Isles of Scilly. At individual sampling sites, Ar ranged from 1.833 at Little Arthur and Old Grimsby Harbour to 2.167 at Broad Ledges Tresco. Allele sizes for all loci at each survey site are shown in Supplementary Table S1. Mean observed heterozygosity, Ho, across all sites was 0.263, with estimates ranging between Ho = 0.229 at Little Arthur and Ho = 0.321 at Broad Ledges Tresco. Expected heterozygosity, He, across all sites was 0.317, ranging from 0.273 at Old Grimsby Harbour to 0.400 at Broad Ledges Tresco. When assessed at the broader scale, observed heterozygosity was significantly lower than expected under Hardy-Weinberg equilibrium (P = 0.005, Table 2), which may indicate some restriction of gene flow between sites. Two individual sites were found to differ significantly from Hardy-Weinberg equilibrium: Little Arthur (P = 0.022) and Old Grimsby Harbour (P = 0.045) (Table 2).

**Table 2:** Estimates of genetic diversity within five populations of *Zostera marina* around the Isles of Scilly, UK, based on six polymorphic microsatellite loci.

Site	Ar	Ho	He	P(HWE)	Fis	Lower	Upper
blt	2.167	0.321	0.400	0.063	0.041	−0.251	0.417
htb	2.000	0.281	0.321	0.871	−0.091	−0.520	0.200
la	1.833	0.229	0.278	0.022	0.025	−0.309	0.460
ogh	1.833	0.244	0.273	0.045	0.023	−0.302	0.608
wbl	2.000	0.238	0.286	0.535	0.018	−0.321	0.320
Overall	1.967	0.263	0.317	0.005	0.105	−0.066	0.275

Site: Sampling site name. Ar: Average number of observed alleles per locus (allelic richness). Ho: Observed heterozygosity. He: Expected heterozygosity; P(HWE). P-values from multilocus tests for Hardy-Weinberg equilibrium (HWE). Fis: Inbreeding coefficient. Lower: Fis lower 95% confidence limit. Upper: Fis upper 95% confidence limit.

There was little evidence of inbreeding at each survey site, with Fis values ranging from −0.096 at Higher Town Bay to 0.044 at Broad Ledges Tresco, and 95% confidence intervals (c.i.s) spanning zero in all cases (Table 2).

### Population genetic structure

Fixation indices comparing observed heterozygosity with that expected under Hardy-Weinberg equilibrium were: Fit = 0.166 (95% c.i.: 0.057, 0.317), Fst = 0.014 (0.002, 0.026), and Fis = 0.105 (−0.066, 0.275). Therefore, overall lower-than-expected heterozygosity (Ho / He = 1 - Fit = 0.834) is primarily underpinned by lower-than-expected heterozygosity associated with inbreeding (Ho / He = 1 - Fis = 0.895) rather than restricted gene flow between sampling sites (Ho / He = 1 - Fst = 0.986). This finding is corroborated by analysis of molecular variance (AMOVA), partitioning population genetic variation into three levels: between the five survey sites, between individuals within sites, and within individuals (Table 3). Total variation was dominated by within-individuals (82.6%) and within-sites levels (15.9%), rather than between well-mixed sites (1.5%).

**Table 3:** Hierarchical analysis of molecular variance (AMOVA) of spatial genetic structure for the five populations of *Zostera marina* based on six microsatellite loci.

Source of Variation	d.f.	Variance component	% Variation	Fixation indices	Lower 95% c.i.	Upper 95% c.i.
Within Individuals	73	1.575	82.6	Fit = 0.166	0.057	0.293
Within Sites	68	0.303	15.9	Fis = 0.105	−0.066	0.275
Between Sites	4	0.029	1.52	Fst = 0.014	0.002	0.026

95% confidence intervals of F-statistics were obtained through bootstrapping over loci with 10,000 iterations.

Pairwise Fst differences between sites ranged from 0.009 for West Broad Ledges vs. Little Arthur to 0.047 for Old Grimsby Harbour vs. Broad Ledges Tresco. Pairwise geographic distances ranged from 955 m between Higher Town Bay and Little Arthur to 4719 m between Broad Ledges Tresco and Little Arthur (Supplementary Table S2). To test the hypothesis of isolation by distance, we performed a Mantel test on the correlation between genetic distance (Fst) and geographic distance (m). This did not support the hypothesis that pairwise genetic divergence correlates with distance (Mantel statistic r = −0.052, P = 0.575).

### Genetic diversity and population trends

**Figure 1:**
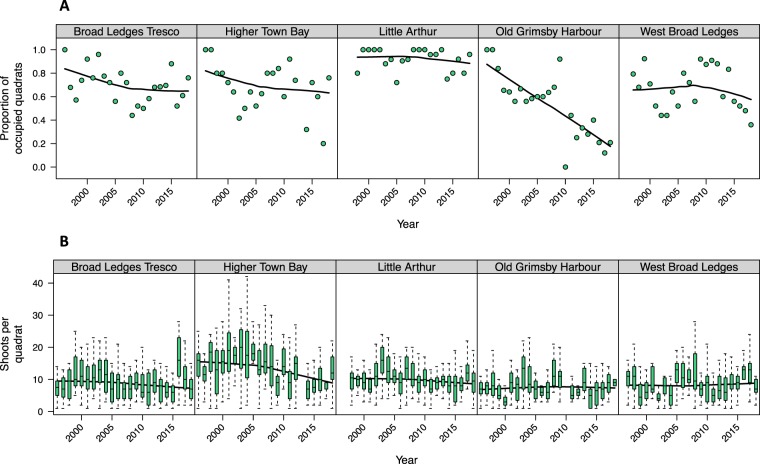
Long-term *Zostera marina* population trends at five sampling sites around the Isles of Scilly, from 1996–2018. (**A**) Proportion of quadrats occupied by seagrass (presence / absence). (**B**) Box-whisker panels for each site summarizing distributions of shoot densities from non-zero quadrats each year. Box midlines show medians. Boxes span interquartile ranges (IQR). Whiskers extend to data points within an additional 1.5 × IQR. Smoothing splines illustrate population trajectories.

We assessed temporal trends in *Z. marina* abundance at two spatial scales, separating out shoot presence / absence in individual quadrats and non-zero shoot counts in occupied quadrats. At the quadrat occupancy level, we recorded long-term declines at several sites, with the most severe at Old Grimsby Harbour (Fig. 1A). Trends in shoot densities from non-zero quadrats are less pronounced but still evident, particularly at Higher Town Bay (Fig. 1B).

Our primary goal here was to assess the spatial scale (within-quadrat shoot density vs. quadrat occupancy) and level of genetic organization (genetic vs. genotypic diversity) that best predict seagrass decline. With only a single snapshot of allelic diversity, this is intended as a guide for future efforts to link population genetics with long-term ecological monitoring, rather than to draw strong conclusions on causality.

As part of a mixture model predicting shoot density and quadrat occupancy, we compared a global model including statistical interactions between allelic diversity (in 2010) and abundance trajectories (1996–2018), then compared this with nested models removing the statistical interactions. This was performed separately with either clonal richness or allele richness as the measure of allelic diversity.

In the case of clonal richness, R, inclusion of even a single snapshot of diversity substantially improved model performance, both for predicting quadrat occupancy (ΔAICc = 45.5) and within-quadrat shoot density (ΔAICc = 4.94). However, in the case of allele richness, Ar, inclusion of genetic information only provided a substantial improvement for quadrat occupancy prediction (ΔAICc = 6.73) but not for within-quadrat shoot density (ΔAICc = 1.32) (Table 4). Fitted surfaces are shown in Supplementary Figure S1.

**Table 4:** Model performance to assess inclusion of a snapshot of allelic diversity in predicting seagrass decline.

**A – Clonal Richness (R)**						
Interaction:	K	AICc	ΔAICc	AICc wt.	Cum. wt.	LL
Both	9	15017.55	—	0.92	0.92	−7499.74
Occupancy	8	15022.49	4.94	0.08	1.00	−7503.22
Density	8	15063.01	45.46	0.00	1.00	−7523.48
Neither	7	15066.92	49.36	0.00	1.00	−7526.44
**B – Allele Richness (Ar)**						
Interaction:	K	AICc	ΔAICc	AICc wt.	Cum. wt.	LL
Both	9	15180.46	—	0.64	0.64	−7581.20
Occupancy	8	15181.78	1.32	0.33	0.97	−7582.87
Density	8	15187.19	6.73	0.02	0.99	−7585.57
Neither	7	15188.29	7.83	0.01	1.00	−7587.13

The full model included statistical interactions between allelic diversity in 2010 and long-term abundance trajectories from 1996–2018. Interactions with allelic diversity were modelled for both within-quadrat shoot density and quadrat occupancy, just quadrat occupancy, just shoot density, or neither (retaining year as a main effect). Allelic diversity was modelled as either A – clonal richness (R) or B – allele richness (Ar). K: Number of model parameters. AICc: Akaike Information Criteria, corrected for small sample size. ΔAICc: Difference in AICc, compared to the best model (-). AICc wt.: AICc weight. Cum. wt.: Cumulative AICc weight. LL: Log-likelihood.

## Discussion

The over-arching aim of this study was to quantify population genetic structure of a relatively isolated and fragmented seagrass population. We undertook this at a location that has been under long-term ecological monitoring, and which is known to be suffering localised declines^11,43,50^. By presenting these findings, we hoped to establish baseline information on genetic diversity and motivate future research into possible causal links between population genetics and abundance trajectories. We performed our research in one of the most extensive and pristine seagrass habitats in the UK, around the Isles of Scilly^49^. Our results demonstrated that a large and sustained seagrass population can persist with low overall levels of genetic diversity. However, we note that even though we only had a single snapshot of the population genetics, a statistical association between lower diversity at that time point and long-term decline was evident.

Seagrass is widely distributed across the shallow waters between the Isles of Scilly^52^; however, there is clear spatial structure and heterogeneity in the occurrence of seagrass across the area^53,54^. Our long-term monitoring has focused on five sampling locations that are separated by distances that are substantially greater than could be spanned by rhizomes growing under the sand^52^. Much of this unpopulated matrix appears suitable for seagrass growth and it is unknown whether seagrass was more continuous historically. In today’s spatial configuration, rafting plant material could easily cross the gaps between our sampling sites. This could result in movement of clonal material – rhizomes that can potentially re-establish at a new location^55,56^ – or of spathes containing viable seeds that can be transported by positively buoyant flowering branches over hundreds of kilometers for weeks to months^57^.

Despite the potential for immigration and metapopulation rescue effects^11,58^ across the archipelago, there is evidence that seagrass is currently declining around the Isles of Scilly, both in terms of local shoot density and fragmentation^50^. This loss is not uniform across the archipelago and, amongst our five sampling sites, we see substantial declines at two sites in particular: Higher Town Bay and Old Grimsby Harbour. The reasons for this are not fully understood and may not be the same at each site. Higher Town Bay is exposed to strong currents but relatively light boat traffic and mooring; whereas Old Grimsby Harbour is the opposite, and mooring is known to be a major cause of localized damage to seagrass^59^. Indeed, the decline manifests primarily as reduced within-quadrat shoot density at Higher Town Bay but reduced quadrat occupancy at Old Grimsby Harbour. We have previously reported differences in population dynamic processes at these two scales^11^ and our new molecular evidence further supports the hypothesis that different processes drive dynamics at these two scales.

In our study, we found seven loci to be monomorphic. Following removal of a further two microsatellites due to linkage disequilibrium, we found an average of 2.0 alleles (maximum = 3) per polymorphic locus. This is compared to between two and eleven alleles per locus (mean = 4.3) in *Z. marina* samples collected in the Wadden Sea, Germany^51^. That study was conducted over a larger area overall, but individual sampling sites within their study were comparable in scale to ours and they reported between site variation to account for only c. 1% of total variation. Additionally, using different markers, allelic richness in twelve *Z. marina* populations across the northern hemisphere ranged from 3.3 to 6.7 (mean = 4.7) alleles per locus^46^. Therefore, we conclude that the *Z. marina* population in the Isles of Scilly shows unusually low allelic richness for this species. It is well known that *Z. marina* across the north Atlantic suffered drastic losses during the 1930s, due to ‘wasting disease’^60,61^. Also, eutrophication has resulted in further, substantial seagrass population declines^14,62^. These types of pressure create the potential for genetic bottlenecks^63^. While other sites across northern Europe may have retained relatively high allelic richness overall through mixing of locally surviving populations, it seems likely that the isolation of the Isles of Scilly has restricted population genetic recovery. The nearest *Z. marina* population to the Isles of Scilly is at Mounts Bay, Cornwall, approximately 50 km distant. While strong east-west currents could transport plant material over that distance, this represents a considerable geographic barrier.

In colonizing coral species, clonal richness declines with increasing physical disturbance^64^. Under extreme high levels of disturbance, the majority of genets can be removed from a location, leading to very low clonal richness^65^. Regular disturbance events have also been linked with low levels of clonal diversity in tropical seagrasses, whereas less frequent disturbance had no effect^66^, suggesting some threshold effect in a species’ ability to resist loss of genetic diversity (see also^39,40^). Old Grimsby Harbour is clearly impacted by damage to the seabed, which provides external forcing of the spatial pattern of seagrass – essentially large holes in previously more uniform vegetation^59^. In contrast, it is not immediately clear what the cause of decline is at Higher Town Bay and a much larger range of patch sizes and spatial complexity is observed there^52^, likely driven by endogenous dynamics^67^.

While allelic richness was low compared to other *Z. marina* populations, we found three out of five sites to be in Hardy-Weinberg equilibrium. This suggests relatively unrestricted gene flow within individual sites, consistent with primarily sexual reproduction through flowering, rather than clonal expansion via rhizomes. Similar findings of Hardy-Weinberg equilibrium in *Z. marina* populations have been reported across northern Europe^46^. As flowering plants, seagrasses can reproduce both sexually, through flowering, and asexually, via rhizome extension. Flowering was to be positively associated with seagrass cover the following year, at the same sampling sites as the current study^43^. Our results corroborate this finding and do not consistently indicate any disruption to the mating system in declining sites. More broadly, when genetic bottlenecks affect populations, higher than expected levels of heterozygosity are predicted^68,69^, in contrast to our findings. If lower than expected levels of allelic richness were the result of a genetic bottleneck driven by historical losses in the last century, the effects of that bottleneck are no longer evident in observed heterozygosity.

Across the whole of the Isles of Scilly, we found significantly less heterozygosity than predicted under Hardy-Weinberg equilibrium. This could be caused by reduced gene flow between sites but other measures of spatial population structure suggest this is not substantial. Nested analysis of molecular variance indicated only 1.5% of total variation attributable to between site differences. This is very similar to levels reported by in the Wadden Sea^51^. Comparing pairwise Fst differences between sites with geographic distances, we found no evidence of isolation by distance. Other studies have identified isolation by distance between *Zostera marina* populations over much larger geographic ranges, e.g., 150–5000 km^70^. Isolation by distance has been reported at the <50 km scale in *Z. marina* but this was only associated with intertidal populations^71^; whereas our populations were sub-tidal. More generally, isolation by distance studies carried out in several seagrass species, notably *Posidonia oceanica*, *Z. marina*, and *Z. noltii* have pointed to this effect occurring over the hundred to thousands of kilometres^40,46,47,70^.

As hypothesised, we did find that lower genotypic diversity is associated with both lower quadrat occupancy and lower within-quadrat shoot density. In addition, lower genotypic diversity was associated with long-term decline at both spatial scales, but substantially more pronounced at the quadrat-occupancy scale. It is important to re-emphasise the limitations of using a single snapshot of population genetics in this study. We are not able to quantify any temporal changes in allelic diversity and our study was not intended to test causal mechanisms or eco-evolutionary feedback processes. However, it is clear that inclusion of some population genetic information improved prediction of abundance trajectories. We hope this provides encouragement for future research in that we have shown ecological and genetic measures are not totally disconnected in this ecosystem.

The value of our current findings is twofold. We show that identifying links between genetic and ecological signals is scale-dependent, providing guidance for future, evidence-based conservation studies. Additionally, we highlight the spatial and temporal scales at which eco-evolutionary dynamics occur, allowing manipulative experiments to test mechanistic hypotheses efficiently.

## Conclusion

The positive association between genetic diversity and population size has been predicted by theory^72^ and supported empirically across a number of species and ecosystems^41^. However, detailed inspection of individual case studies has led to equivocal and scale-dependent findings. Seagrasses often exist as natural monocultures and, therefore, make ideal model systems to test ecological and population genetic hypotheses. This model has proved informative in both population dynamic^11,73,74^ and population genetic studies^39,66^ but these are rarely linked. In this study, we estimated a suite of common genetic and genotypic diversity parameters amongst *Zostera marina* populations exhibiting a range of long-term population trajectories. Overall, we found evidence of low allelic diversity, as well as declining abundance over a decadal timescale. Integration of these data sources highlighted that genotypic, rather than genetic, diversity may be the appropriate level of genetic organization and that quadrat occupancy, rather than within-quadrat shoot density, may be the appropriate spatial scale to develop future research into causal links. This adds to the growing body of evidence that identifying genetic and ecological indicators underpinned by eco-evolutionary mechanisms is essential for marine spatial planning, conservation, and management. Our findings also help to address urgent calls to develop integrated ecological and genetic frameworks to inform evidence-based management of this globally important but widely threatened group^75^, as well as other vegetative ecosystems.

## Materials and Methods

### Sample collection

**Figure 2:**
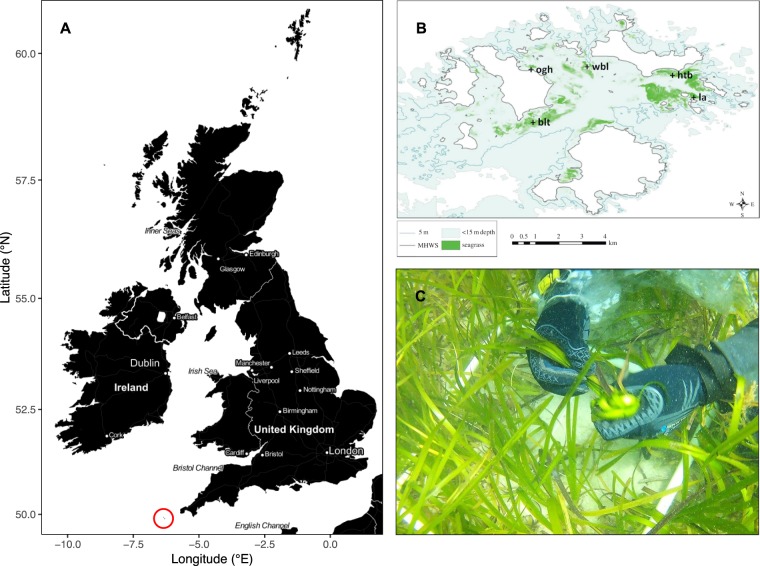
Distribution and sampling of seagrass meadows (*Z. marina*) throughout the Isles of Scilly, UK. (**A**) Position of the Isles of Scilly (red circle), relative to the UK and Ireland. (**B**) Locations of the five long-term monitoring sites: Broad Ledges Tresco (blt), Higher Town Bay (htb), Little Arthur (la), Old Grimsby Harbour (ogh), and West Broad Ledges (wbl). (**C**) Representative illustration of seagrass condition and quadrat sampling.

The Isles of Scilly, UK, supports a substantial but spatially fragmented population of eelgrass, *Zostera marina* (Fig. 2). As part of a long-term, on-going program of annual monitoring since 1996^50^, we collected leaf samples from five sites around the archipelago in July 2010: Broad Ledges Tresco (“blt”: 49° 56.396′ N, 06° 19.773′ W), Higher Town Bay (“htb”: 49° 57.382′ N, 06° 16.336′ W), Little Arthur (“la”: 49° 56.945′ N, 06° 15.914′ W), Old Grimsby Harbour (“ogh”: 49° 57.603′ N, 06° 19.663′ W), and West Broad Ledges (“wbl”: 49° 57.407′ N, 06° 18.774′ W) (Fig. 2).

At each of the five long-term monitoring sites, we randomly placed 25 quadrats (0.25 m × 0.25 m) within 30 m of a central datum, using pre-assigned coordinates each year^73^. Quadrats are not left *in situ* between years so do not overlie those in previous years. Shoot density was recorded within each 0.0625 m^2^ quadrat. Due to the fragmented nature of the seagrass, some quadrats were located on bare substrate, allowing an estimate of broader within-site coverage, or spatial heterogeneity. In 2010, we collected leaf samples from 20 quadrats at four of the five sites. Due to extremely low seagrass coverage at Old Grimsby Harbour that year, we instead placed 16 quadrats haphazardly but at least 2 m apart on areas of seagrass. This represents a similar scale of separation to our other sampling sites, as well as other studies (e.g.,^51^), and *Zostera marina* clones have been estimated at less than 2 m across^76^. This combination of random bearing and distance sampling where possible, with haphazard sampling > 2 m apart where not, is almost identical to that of ^66^.

At each quadrat station, we collected approximately 2–3 cm of fresh tissue from a single leaf, which was transported to the laboratory for further analysis. Samples were then washed using freshwater and raked to remove epiphytic algae. Leaf samples were dried at −109 °C for 24 to 48 h using a SCANVAC CoolSafe 110–4 Pro and then stored frozen (−20 °C) until DNA was extracted from leaf tissue.

### Molecular methods

Frozen samples were ground with a Precellys Ceramic Kit 1.4/2.8 mm. Genomic DNA was extracted using Qiagen DNeasy Plant Mini Kits following the manufacturer’s instructions. Purity and concentration of DNA was determined by the A260/280 nm absorbance using an Implen NanoDrop 2000 Spectrophotometer and stored at −20 °C.

We used an existing panel of 15 polymorphic microsatellite markers^51^. Microsatellite loci were amplified by polymerase chain reaction (PCR). Each forward primer was labelled with a fluorescent dye (FAM or HEX) to allow multiplexing, labelled and pooled as per^51^.

PCR amplification was accomplished according to the manufacturer’s protocols using Qiagen Type-it Microsatellite PCR kits. Reactions were performed in a reaction volume of 15 μl containing 7.5 μl of 2x Type-it Multiplex PCR Master Mix, 1.5 μl of 10x primer mix, approximately 4.2–5.9 μl RNase-free water, and approximately 0.1–1.8 μl of template DNA. PCR cycling conditions comprised an initial denaturation heat activation at 95 °C for 5 min, followed by 3-step cycling of 95 °C for 30 s (denaturation), 57–63 °C for 90 s (annealing), and 72 °C for 30 s (extension) for 28 cycles followed by a terminal extension phase at 60 °C for 30 min.

PCR products were sent to the Institute of Biological, Environmental, and Rural Sciences (IBERS), Aberystwyth University, UK, to obtain read lengths.

### Allele scoring

Microsatellite DNA fragment lengths were used as the basis of allele scoring. This was achieved using the R package Fragman, version 1.0.9, automatically with default settings and confirmed through manual inspection^77^. We visualized the scoring and binning of microsatellite alleles using the R package MsatAllele, version 1.0^78^. This permitted the identification of monomorphic and polymorphic loci, as well as providing a further check of allele identifiability at the population level. Presence of null alleles was assessed using FreeNA software^79^.

To verify the independence of loci, we tested for linkage disequilibrium using GENEPOP, version 4.2^80^. Pairwise comparisons between all loci (n = 8, following removal of seven monomorphic loci) were made using likelihood ratio tests with Benjamini and Yekutieli false discovery rate correction^81^.

### Population genetic analysis

We assessed the probability of two or more samples coming from independent reproductive events (rather than resampling of the same genet) by identifying potential resampled individuals using P_SEX_^82^. This identified 23 of our 96 samples as having an unacceptably low probability of being from independent reproductive events (P < 0.05), i.e., clonal replicates, and these were removed for subsequent analysis.

We tested whether differing sample sizes between sites introduced bias by comparing the observed number of multilocus genotypes (MLG) at each survey site, with the number of expected multilocus genotypes (eMLG – the number of multilocus genotypes predicted using the lowest number of samples) at each site, using the R package Poppr, version 2.8.1^83^. We also calculated a common measure of genotypic diversity: clonal richness, R = (MLG − 1) / (N − 1), where N is the sample size^84^.

To evaluate microsatellite diversity in the Isles of Scilly eelgrass, we calculated the observed number of alleles (Ar – allelic richness), estimates of observed (Ho) and expected (He – assuming Hardy-Weinberg equilibrium) heterozygosity, and Wright’s fixation indices (Fis, Fst, and Fit) in the five populations using the R package Poppr, version 2.8.1^83^.

To assess the spatial population genetic structure of *Z. marina* around the Isles of Scilly, a hierarchical analysis of molecular variance (AMOVA) was performed using GenoDive, version 2.0b23^85^. Nested variance components were: between the five survey sites, between individuals within sites, and between alleles within individuals. Pairwise estimates of Fst were also calculated^86^, with 95% confidence intervals obtained through bootstrapping (1000 iterations).

We explored the potential relationship between genetic and geographic distance by comparing the pairwise Fst differences with distances between sites^87^. Pairwise Euclidean distances were calculated using the R package Geosphere, version 1.5–7^88^. We tested the statistical significance of correlation between genetic and geographic distances using a Mantel test with 10,000 permutations^89^.

### Population trajectories

We tested hypotheses on the relationship between genetic diversity and long-term population abundance trajectories using a Generalised Linear Modelling (GLM) framework. We assessed the effects of genetic diversity (Ar – allelic richness) and genotypic diversity (R – clonal richness) on population abundance using separate GLMs. *Z. marina* abundance was estimated from shoot counts in replicate quadrats at each of five survey sites over 23 years. These zero-inflated data were partitioned into presence / absence within individual quadrats and counts in non-zero quadrats, then assessed simultaneously using a mixture model, implemented as a GLM statistical model^90^. Presence / absence was modelled as a binomial distribution; non-zero count data were modelled as a zero-truncated negative binomial distribution. Ecological monitoring year (1996–2018) and allelic diversity (either Ar or R) in 2010 were fitted as fixed effects, both as main effects and through their statistical interaction.

Where statistical analysis was performed using R, this was run in version 3.5.1^91^.

## Supplementary information

Supplementary Information

## Data Availability

The datasets generated and analysed during the current study are available from the corresponding author on reasonable request.
