# Supplementary material for: Low genotypic diversity and long-term ecological decline in a spatially structured seagrass population
Source: Sci Rep. 2019 Dec 5;9:18387. doi: 10.1038/s41598-019-54828-1 (PMC6895181; doi:10.1038/s41598-019-54828-1)
Supplement: Supplementary file 1 — Supplementary Information [file 41598_2019_54828_MOESM1_ESM.pdf]

Low genotypic diversity and long-term ecological decline in a spatially structured seagrass population

Nahaa M. Alotaibi[1,2], Emma J. Kenyon[3], Kevan J. Cook[4], Luca Börger[1], and James C. Bull[1]*

Supplementary Information

Table S1. Observed allele sizes in our five sampling sites around the Isles of Scilly, U.K.: Broad Ledges Tresco (blt), Higher Town Bay (htb), Little Arthur (la), Old Grimsby Harbour (ogh), and West Broad Ledges (wbl).

	CL559Contig1	CL202Contig1	CL805Contig1	CL172Contig1	ZMC05062	ZME05315
blt	173, 177	202, 215	188, 190, 194	193, 197	98, 102	180, 182
htb	173, 177	202, 215	188, 190, 194	193, 197	98	180, 182
la	173	202, 215	188, 194	193, 197	98, 102	180, 182
ogh	173	202, 215	188, 194	193	98, 100, 102	180, 182
wbl	173, 177	202, 215	188, 194	193, 197	98, 102	180, 182

Table S2. Pairwise distance matrix between five *Zostera marina* meadows in the Isles of Scilly, UK. Upper triangle shows genetic distance (Fst). Lower triangle shows geographic distance (m) between sampling sites.

	blt	htb	la	ogh	wbl
blt	-	0.030	0.024	0.047	0.031
htb	4493	-	0.028	0.038	0.019
la	4719	955	-	0.026	0.009
ogh	2243	3992	4639	-	0.027
wbl	2223	2911	3520	1122	-

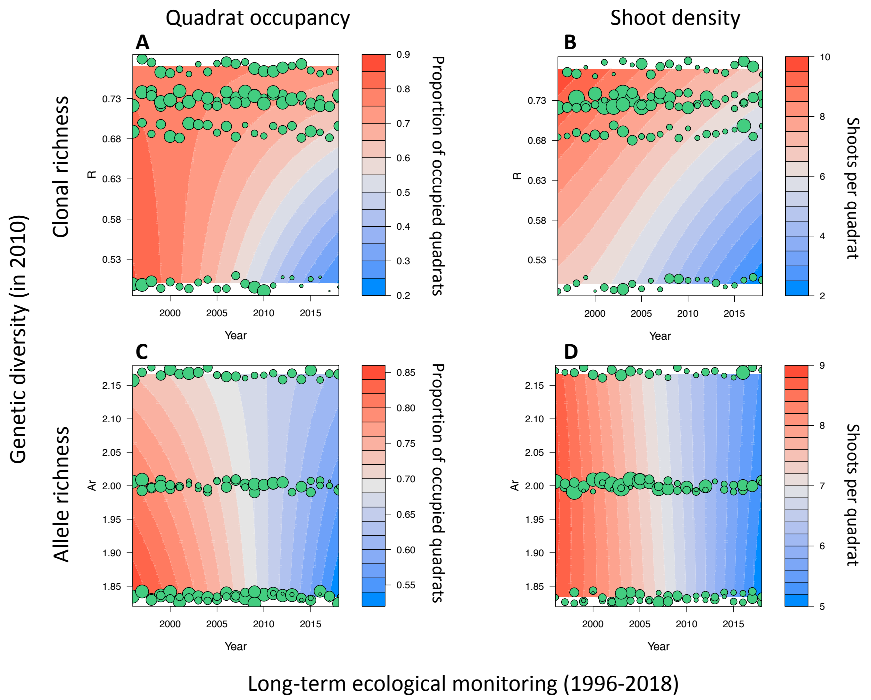

Figure S1. The association between genetic diversity and long-term population trends in *Zostera marina*. (A) Clonal richness (R) against quadrat occupancy. (B) Clonal richness (R) against non-zero shoot density. (C) Allele richness (Ar) against quadrat occupancy. (D) Allele richness (Ar) against non-zero shoot density. Colour scale represents fitted values. Point sizes indicate median values from empirical data. A small amount of jitter is applied to the y-coordinate for illustrative purposes.
